# Neutralizing Antibodies against *Plasmodium falciparum* Associated with Successful Cure after Drug Therapy

**DOI:** 10.1371/journal.pone.0159347

**Published:** 2016-07-18

**Authors:** Yun Shan Goh, Kaitian Peng, Wan Ni Chia, Anthony Siau, Kesinee Chotivanich, Anne-Charlotte Gruner, Peter Preiser, Mayfong Mayxay, Sasithon Pukrittayakamee, Kanlaya Sriprawat, Francois Nosten, Nicholas J. White, Laurent Renia

**Affiliations:** 1 Singapore Immunology Network, Agency for Science, Technology and Research, Singapore, Singapore; 2 Department of Microbiology and Immunology, Yong Loo Lin School of Medicine, National University of Singapore, Singapore; 3 School of Biological Sciences, Nanyang Technological University, Singapore, Singapore; 4 Faculty of Tropical Medicine, Mahidol University, Bangkok, Thailand; 5 Lao-Oxford-Mahosot Hospital-Wellcome Trust Research Unit, Mahosot Hospital, Vientiane, Laos; 6 Shoklo Malaria Research Unit, Mahidol-Oxford Tropical Medicine Research Unit, Faculty of Tropical Medicine, Mahidol University, Mae Sot, Thailand; 7 Centre for Tropical Medicine and Global Health, Nuffield Department of Medicine, University of Oxford, Oxford, United Kingdom; 8 Mahidol Oxford Research Unit, Faculty of Tropical Medicine, Mahidol University, Bangkok, Thailand; Johns Hopkins Bloomberg School of Public Health, UNITED STATES

## Abstract

An effective antibody response can assist drug treatment to contribute to better parasite clearance in malaria patients. To examine this, sera were obtained from two groups of adult patients with acute falciparum malaria, prior to drug treatment: patients who (1) have subsequent recrudescent infection, or (2) were cured by Day 28 following treatment. Using a *Plasmodium falciparum* antigen library, we examined the antibody specificities in these sera. While the antibody repertoire of both sera groups was extremely broad and varied, there was a differential antibody profile between the two groups of sera. The proportion of cured patients with antibodies against EXP1, MSP3, GLURP, RAMA, SEA and EBA181 was higher than the proportion of patients with recrudescent infection. The presence of these antibodies was associated with higher odds of treatment cure. Sera containing all six antibodies impaired the invasion of *P*. *falciparum* clinical isolates into erythrocytes. These results suggest that antibodies specific against EXP1, MSP3, GLURP, RAMA, SEA and EBA181 in *P*. *falciparum* infections could assist anti-malarial drug treatment and contribute to the resolution of the malarial infection.

## Introduction

Malaria is a major public health problem with high mortality and morbidity. The World Health Organization estimates 214 million clinical episodes of malaria, and 438,000 deaths in 2015 [1]. The disease is caused by the protozoan parasite *Plasmodium* and transmitted by *Anopheles* mosquitoes. Five *Plasmodium* species, namely *P*. *falciparum*, *P*. *vivax*, *P*. *malariae*, *P*. *ovale* and *P*. *knowlesi*, are capable of infecting humans. The vast majority of malaria-related mortality is caused by *P*. *falciparum* [2]. Naturally acquired immunity can develop in individuals living in malaria-endemic regions and this naturally acquired immunity to malaria has been shown to protect these individuals against malaria [3–5]. Passive transfer of sera from chronically exposed individuals eliminated blood parasites in *P*. *falciparum*-infected patients [6, 7]. However, the protection mediated is not always sterilising against infection by all isolates/strains of *Plasmodium* and in all individuals living in malaria-endemic regions. This is likely because the development of naturally acquired protective immunity requires multiple exposures over a long period of time to attain a broad repertoire of protective antibodies [3–5].

In this study, we hypothesized that an effective antibody response can predispose infected patients receiving drug therapy to a better disease resolution by assisting in parasite clearance. In areas where drug resistance results in inefficient parasite clearance, naturally acquired immunity with effective *P*. *falciparum*-specific antibody response could help to contain parasite levels and increase drug efficacy by facilitating parasite elimination. To investigate the contribution of immunity to treatment outcomes in patients, we examined the antibody responses in individuals living in endemic areas in Thailand by comparing the sera from patients who have subsequent recrudescent infection, or were cured following drug treatment. Using a *P*. *falciparum* antigen library, we studied the antigen-specific antibody profile of these sera and aimed to identify an antibody profile that could predispose both severe and uncomplicated malaria patients to better disease resolution. We also studied how these sera affect merozoite invasion to examine how the immunity can aid drug treatment to contribute to treatment success in the Thai patients.

## Materials and Methods

### Ethics

For the human sera used, the study was approved by Ethics Committee of the Faculty of Tropical Medicine, Mahidol University, Bangkok, Thailand and performed in accordance with approved guidelines and regulations [8]. Written informed consent was obtained from all patients prior to the study. For the *P*. *falciparum* field isolates used, infected human blood samples were collected under the ethical guidelines in the approved protocols: OXTREC 027–025 (University of Oxford, Centre for Clinical Vaccinology and Tropical Medicine, UK) and MUTM 2008–215 from Ethic committee of Faculty of Tropical Medicine of Mahidol University.

### Study sera

Sera were obtained from adult Thai patients with acute falciparum malaria, admitted to the Hospital for Tropical Diseases, Bangkok, Thailand and remained for 28 days, at admission prior to drug treatment. The sera were as previously studied [8]. The sera were divided into two groups based on their subsequent treatment status: (1) patients who have subsequent recrudescent infection (*n =* 28), (2) patients who were cured by Day 28 following treatment (*n =* 40). Plasma samples from patients who developed subsequent recrudescent infections (without reexposure) after antimalarial treatment were matched for age, therapeutic regimen, and disease severity and analyzed with samples from those who were cured. The cured patients were previously untreated patients entered in prospective antimalarial drug efficacy studies between November 1998 and April 1999, and were selected prior to antibody analysis.

The recrudescent sera were obtained from 13 severe malaria patients and 15 uncomplicated malaria patients, while the cured sera were obtained from 13 severe malaria patients and 27 uncomplicated malaria patients. Due to serum amount availability, the recrudescent serum set was smaller than the previous study [8] (with 15 from uncomplicated malaria patients, instead of 27). In the serum set used in this study (Table 1), age of the patients did not differ significantly between the different groups. There was significant difference in the parasite clearance times between the cured and recrudescent patients with uncomplicated malaria (*P* = 0.0046, by Mann-Whitney *U*-test). Parasite and fever clearance times at recrudescence differ significantly between severe and uncomplicated malaria patients (*P* = 0.0019 and *P* = 0.02 respectively, by Mann-Whitney *U*-test). The geometric mean of the admission parasite density did not differ significantly between the different groups. All patients were treated with short-acting antimalarial drugs (artesunate alone or in combination with azithromycin or artemether plus lumefantrine). The proportion of the cured group that received artesunate monotherapy was 57.5% (23/40), which was comparable to the 53.6% (15/28) of the recrudescent group receiving monotherapy.

**Table 1:** Patients’ serum groups.

	Cure (*n =* 40)			Recrudescence (*n =* 28)		
Variable	Severe malaria (*n =* 13)	Uncomplicated malaria (*n =* 27)	Total	Severe malaria (*n =* 13)	Uncomplicated malaria (*n =* 15)	Total
**Age, years (mean S.D.)**	23.8 ± 8.4	23.1 ± 5.4	23.9 ± 6.5	24.1 ± 7.6	25.7 ± 9.5	24.5 ± 7.7
**Parasite Clearance Time, hr (mean ± S.D.)**	43.1 ± 12.2	43.8 ± 8.1	44.4 ± 10	44.6 ± 8.5	60.1 ± 17.4	51.6 ± 16.0
**Fever Clearance Time, hr (median, range)**	37, 18–156	28, 0–144	32, 0–156	100, 26–148	36, 8–83	39, 8–148
Admission parasite density/uL (geometricmean, range)*	5.11, 3.72–5.95	4.26, 2.78–5.23	4.53, 2.78–5.95	4.76, 3.92–5.90	4.49, 3.04–5.30	4.62, 3.04–5.90
**Proportion treated with Artesunate alone, %**	100.0	37.0	57.5	69.2	40	53.6

*Log_10_ scale.

### *P*. *falciparum* antigen library

The *P*. *falciparum* antigen library was as previously described [9]. Briefly, nucleotide sequences encoding for *P*. *falciparum* antigens were amplified *via* PCR, using either *P*. *falciparum* 3D7 genomic DNA or RNA as template, and cloned into the pDisplay vector (Invitrogen). The resultant plasmids were then transfected into HEK293 cells using lipofectamine 2000 (Invitrogen) for surface expression of the *P*. *falciparum* antigens. The *P*. *falciparum* antigen has a hemagglutinin (HA) tag at the N-terminal of the antigen and a myc tag at the C-terminal of the antigen, allowing detection of expression level of the *P*. *falciparum* antigen using an anti-HA (Sigma) or anti-myc (Miltenyi Biotec) antibodies.

### Antibody profiling of patients’ serum response

Determination of the antibody profile of the patients’ was as previously described [9]. Briefly, transfected cells, expressing *P*. *falciparum* antigens on the cell surface, were first incubated with human serum (diluted 1:100 in FACS blocking buffer (10% FBS in PBS)) on shaking. The cells were then incubated with a double stain, consisting of Alexa Fluor 488-coupled secondary antibodies (Invitrogen; diluted 1:500) and propidium iodide (PI; diluted 1:2500) on shaking. Cells were read on Accuri C6 (BD Biosciences) and analyzed using FlowJo (Tree Star). Parallel to the determination of presence of specific antibodies in the patients’ sera, the transfected cells were also stained separately with rabbit anti-myc or anti-HA antibodies (diluted 1:100) to determine the transfection efficiency.

The analysis followed the below five steps: (1) determining the transfection efficiency of the Pf antigen-transfected cells: the proportion of cells that are transfected and expressing the Pf antigen, which is also defined as Alexa Fluor 488-positive and PI-negative labelling (PI-negative labelling indicates live cells) (S1A and S1B Fig); (2) determining the presence of *P*. *falciparum*-specific antibody response in each serum in the two group of patients’ sera (cured or recrudescent patients): gated on negative controls (Pf antigen-transfected cells with serum from healthy volunteers, S1C Fig; and non-transfected cells with patient sera, S1D Fig) and defined by Alexa Fluor 488-positive and PI-negative labelling (S1E Fig); (3) quantifying the *P*. *falciparum*-specific antibody reactivity by normalizing the antibody response to transfection efficiency: the proportion of cells with bound sera antibodies (from step 2) was divided by the transfection efficiency (from step 1), and then multiplied by 100 to obtain a percentage; (4) determining if the *P*. *falciparum*-specific antibody response was positive: *P*. *falciparum*-specific antibody response was defined as positive when the antibody response (from step 3) was at a minimum of 10%; (5) determining the proportion of cured patients’ sera and recrudescent patients’ sera with a positive *P*. *falciparum*-specific antibody response: defined as the ratio of the number of patients with a positive *P*. *falciparum*-specific antibody response (> 10%; from step 4) to the total number of patients in the group (either cured or recrudescent patient group), expressed as a percentage.

### *Plasmodium* culture

For merozoite invasion assays, 12 *P*. *falciparum* isolates (the laboratory strain 3D7, and 11 field isolates) were used. The 11 isolates were collected between in 2009 and 2010 from malaria patients attending the Shoklo Malaria Research Unit (SMRU) clinics, Mae Sot region of Tak Province in northwestern Thailand. They were from patients with no prior antimalarial therapy and with microscopically confirmed *P*. *falciparum*. After written consent, blood samples were collected by venepuncture in 5-ml-volume lithium heparinized tubes. They were then transported to the laboratory at SMRU and processed to remove platelets and leukocytes within 5 hours of collection [10]. Cryopreservation was performed using Glycerolyte 57 (Baxter).The parasites were cultured in vitro using RPMI-HEPES medium pH 7.4 supplemented with 50 μg/ml hypoxanthine, 25 mM NaHCO3, 2.5 μg/ml gentamicin and 10% serum at 37°C, 5% CO2. For 3D7 culture, Albumax was used as serum. For field isolates, human serum from malaria-negative healthy donors was used.

### Merozoite invasion inhibition assay

Merozoite invasion inhibition assays were performed as previously described [9]. Briefly, MACS-sorted *P*. *falciparum* late-trophozoite/schizonts were added to CFSE-stained red blood cells (RBCs; stained at a final concentration of 30 μM CFSE) at a final parasitemia of 1% and hematocrit of 2%. Human sera were added at a 1:50 dilution. The mixture was incubated at 37°C with 5% CO_2_. After 24 hr, cultures were stained with Hoechst 33342 (Sigma; diluted 1:100). Parasitemia was determined by flow cytometry and newly invaded RBCs were defined as CFSE and Hoechst double positive RBCs. RBC invasion inhibition efficiency was defined as the ratio of the subtraction of parasitemia in the test well (patients sera) from the parasitemia in the control well (normal human serum) to the parasitemia in the control well, expressed as a percentage. Six pools of sera were analysis for merozoite invasion inhibition–(1) 6Ab +ve: sera containing antibodies specific for EXP1, MSP3, GLURP, RAMA, PfSEA, EBA181 (*n =* 14); (2) cured 5Ab +ve sera: sera from cured patients containing EXP1, MSP3, GLURP, RAMA, PfSEA (*n =* 14); (3) 6Ab–ve: sera without no detected antibodies against EXP1, MSP3, GLURP, RAMA, PfSEA, EBA181 (*n =* 6); (4) recrud 5Ab +ve: sera from recrudescent patients containing EXP1, MSP3, GLURP, RAMA, PfSEA (*n =* 7); (5) pooled cured: sera from all cured patients (*n =* 40); (6) pooled recrud: sera from all recrudescent patients (*n =* 28).

### Statistical analysis

For comparison of parasite and fever clearance time between different serum groups, and comparison of invasion inhibition efficiency between different serum pools, statistical analysis was performed using Mann-Whitney *U*-test.

For serum reactivity against *P*. *falciparum* antigens, comparisons of proportions between different groups of sera were performed using Fisher’s exact test on contingency table, where *p* value < 0.05 was considered to be significant. Both non-adjusted and adjusted *p* values (adjusted for multiple comparisons using Bonferroni correction) were shown.

Odds ratio was calculated using GraphPad Prism. It is defined as the odds that cure will occur in the presence of specific antibodies, compared to the odds of a cure occurring in the absence of specific antibodies.

For merozoite invasion inhibition assay, comparisons of data from different groups of sera were performed using the Mann-Whitney *U*-test, where *p* value < 0.05 was considered to be significant. *P* value has been corrected for multiple comparisons using Bonferroni correction.

## Results

### Antibody repertoire of sera obtained from exposed individuals

To investigate the contribution of naturally acquired immunity to treatment outcomes in patients, we first examined if we could identify an antibody profile that could predispose both severe and uncomplicated malaria patients to treatment success. We compared sera from patients (both severe and uncomplicated malaria patients) who (1) have subsequent recrudescent infection, or (2) were cured by Day 28 following drug treatment (Table 1). Both groups of sera were obtained from adult Thai patients with acute falciparum malaria, prior to drug therapy [8].

Using our *P*. *falciparum* antigen library, we examined the antibody repertoire of the two groups of sera. The *P*. *falciparum* antigen library, cloned in the pDisplay vector, consisted of 146 different constructs encoding for different *P*. *falciparum* antigens and was transfected into HEK293 cells for cell surface expression. The antigens represented in our library were specifically selected based on studies that reported expression on the surface of the parasite or infected erythrocytes and association studies that postulated antigens to be potential vaccine candidates. For this study, we focused on 84 antigens that are expressed during the blood stage (either restricted to the blood stage or across different stages), representing 60 different *P*. *falciparum* genes.

The antibody repertoire of the two groups of sera was extremely broad and varied (Table 2; S1 Table). Using pooled sera from either cured or recrudescent patients, we screened for reactivity against our library of 84 antigens (Table 2). The pooled cured sera had a positive IgG response against 48 out of 84 antigens (57.14%) and a positive IgM response against 35 out of 84 antigens (41.67%). The pooled recrudescent sera had a positive IgG response against 39 out of 84 antigens (46.43%) and a positive IgM response against 26 out of 84 antigens (30.95%).

**Table 2:** Proportion of screened antigen library represented in patients’ sera.

	Cured patients		Recrudescent patients	
	IgG	IgM	IgG	IgM
Proportion of screened antigen library represented in sera (%)	48/84 (57.14)	35/84 (41.67)	39/84 (46.43)	26/84 (30.95)

### Differential antibody profile between cured and recrudescent patients

We proceeded to study if the cured patients had a differential antibody profile that might contribute to more efficient parasite elimination. Using individual serum to screen against the *P*. *falciparum* antigen library, we determined the proportion of patients in each group that contained antibodies against each of the antigens in the library (S2 Table).

There was a differential IgG profile between cured and recrudescent patients. Out of the 84 antigens screened, higher proportion of cured patients had specific IgG against EXP1 (amino acid 102–162), MSP3, GLURP, RAMA, SEA and EBA181 in their pre-drug sera, as compared to recrudescent patients (Table 3; unadjusted *p*<0.05). However, after the *p* values were adjusted for multiple comparisons, we found specific IgG against EXP1, MSP3, GLURP, RAMA statistically differential between the cured and the recrudescent patients, but not SEA and EBA181 (Table 3). The difference in the proportion of cured patients and recrudescent patients with anti-RAMA IgG was the highest, at 53%, and the difference in the proportion of cured patients and recrudescent patients with anti-EBA181 IgG was the lowest, at 26%. We did not observe a statistically differential IgM profile between the cured and the recrudescent patients. Low levels of specific IgM could be due to high seroconversion as the patients might have been previously exposed to the parasite [8]. When the sera were stratified based on disease severity (severe or uncomplicated malaria), we also observed a similar pattern in both severe and uncomplicated malaria groups, where there was a consistently higher proportion of cured patients with specific IgG against EXP1, MSP3, GLURP, RAMA, SEA and EBA181 in their pre-drug sera, as compared to recrudescent patients (S3 Table).

**Table 3:** Differential antibody profile of sera obtained from cured and recrudescent patients.

			Specific IgG			Specific IgM		
Gene name	plasmodb	TE* (%)	Cured patients, %	Recrudescent patients, %	*p* value (adjusted**)	Cured patients, %	Recrudescent patients, %	*p* value
EXP1	PF3D7_1121600	87	95	65	0.0003 (0.0249)	57.14	46.43	NS
MSP3	PF3D7_1035400	92	95	35	0.0001 (0.0084)	46.43	28.57	NS
GLURP	PF3D7_1035300	91	97.5	77.5	0.0005 (0.0411)	64.29	35.71	NS
RAMA	PF3D7_1035300	78	85	22.5	0.0001 (0.0084)	32.14	25	NS
PfSEA	PF3D7_1021800	42	85	25	0.0224 (0.8509)	57.14	25	NS
EBA181	PF3D7_0102500	83	55	22.5	0.0326 (0.9382)	28.57	28.57	NS

*TE: Transfection efficiency.

** *p* value adjusted for multiple comparisons using Bonferroni correction.

To examine if the presence of specific antibodies was associated with better disease resolution following drug treatment, we determined the odds of subsequent cured infection in patients with specific antibodies against EXP1, MSP3, GLURP, RAMA, SEA and EBA181 (Table 4). Presence of specific IgG against each of the six antigens was associated with higher odds (<1) of subsequent cured infection. We then examined the odds of subsequent cured infection in patients with specific antibodies against the four antigens that were found to be statistically differential between cured and recrudescent patients (EXP1, MSP3, GLURP, RAMA; adjusted *p* value<0.05). However, the analysis was limited by number of sera (cured and recrudescent) containing specific antibodies against only the four antigens (*n =* 2). Hence, we have extended our analysis of the odds of cured infection in patients and all subsequent analysis to sera containing specific antibodies against the six antigens EXP1, MSP3, GLURP, RAMA, SEA and EBA181). The odds were the highest when specific IgG against all six antigens was all present. On the contrary, only the presence of IgM against EXP1, MSP3, and GLURP was associated with higher odds of cured infection. When we analyzed the data based on sera containing specific antibodies (IgG and/or IgM), we observed a similar pattern as that observed with specific IgG.

**Table 4:** Odds ratio of cured infection in the presence of specific antibody.

		Odds ratio of cured infection in the presence of specific Ab (95% CI)		
Gene name	Plasmodb	IgG	IgM	IgG and/or IgM
EXP1	PF3D7_1121600	14.25 (2.856–71.1)	2.143 (0.7986–5.75)	25.24 (3.013–211.4)
MSP3	PF3D7_1035400	21.92 (4.406–109.1)	1.346 (0.4728–3.833)	39 (4.685–324.7)
GLURP	PF3D7_1035300	21.67 (2.573–182.4)	6.2 (2.123–18.11)	15.6 (1.821–133.7)
RAMA	PF3D7_1035300	11.96 (3.691–38.78)	0.871 (0.2806–2.704)	10.82 (3.24–36.13)
PfSEA	PF3D7_1021800	4.25 (1.351–13.37)	1 (0.3277–3.051)	4.529 (1.356–15.13)
EBA181	PF3D7_0102500	3.382 (1.205–9.497)	0.7258 (0.2401–0.194)	3.515 (1.266–9.758)
4Ag*		3.701 (0.1709–80.17)	-	3.701 (0.1709–80.17)
6Ag**		31.19 (1.77–549.6)	2.165 (0.08499–55.13)	31.19 (1.77–549.6)

*4Ag: EXP1, MSP3, GLURP, RAMA.

**6Ag: EXP1, MSP3, GLURP, RAMA, PfSEA, EBA181.

### Sera containing specific *P*. *falciparum* antibodies inhibit merozoite invasion

We hypothesized that the association between specific EXP1, MSP3, GLURP, SEA and EBA181 IgG and higher odds of having a subsequent cured infection might be due to a contributory role in the containment of the parasite levels in the blood. This could be a result of specific IgG against EXP1, MSP3, GLURP, RAMA, SEA and EBA181 inhibiting *P*. *falciparum* merozoite invasion.

**Fig 1:**
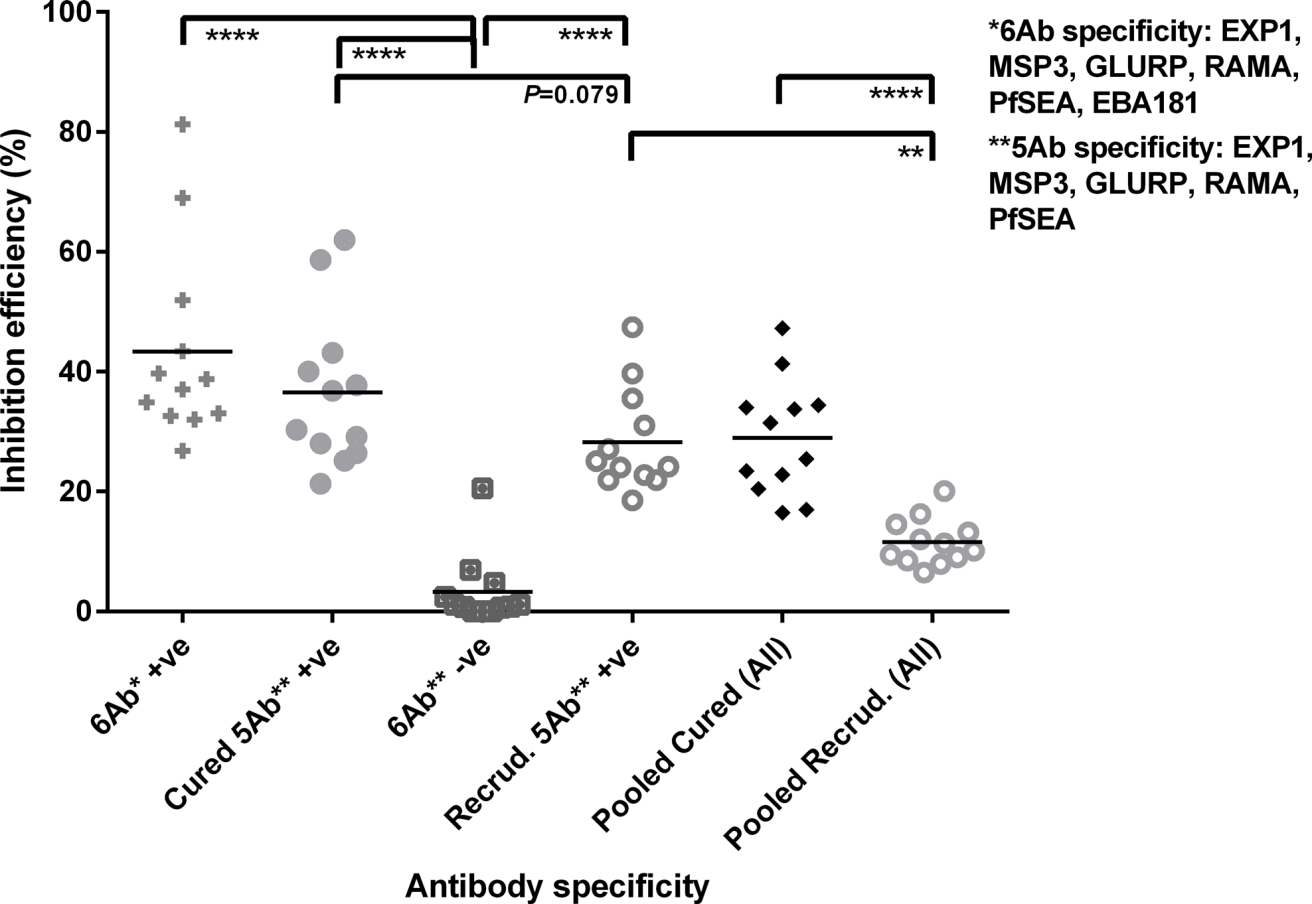
Antibody-mediated inhibition of Pf RBC invasion. RBC invasion inhibition efficiency, mediated by the human patients sera, was defined as the ratio of the subtraction of parasitemia in the test well (test patients sera) from the parasitemia in the control well (normal human serum) to the parasitemia in the control well, expressed as a percentage. 6Ab +ve refers to sera (both cured and recrudescent sera) containing specific antibodies against all six antigens (*n =* 14); Cured 5Ab +ve refers to cured sera containing specific antibodies against the five antigens (*n =* 14); 6Ab–ve refers to sera (both cured and recrudescent sera) with absence of specific antibodies against the six antigens (*n =* 6); Recrudescent 5Ab +ve refers to recrudescent sera containing specific antibodies against the five antigens (*n =* 7). Pooled cured refers to sera pooled from all cured patients (*n =* 40) and pooled recrudescent refers to sera pooled from all recrudescent patients (*n =* 28). Each point in graph represents one *P*. *falciparum* isolate (*n =* 12). Horizontal line in graph represents mean inhibition efficiency between the 12 *P*. *falciparum* isolates. * denotes *p* <0.05; ** denotes *p* <0.01; *** denotes *p* <0.001; **** denotes *p* <0.0001, by Mann-Whitney *U*-test (adjusted for multiple comparison using Bonferroni correction).

From our odds ratio data, we have observed that sera containing IgG against all six antigens (EXP1, MSP3, GLURP, RAMA, SEA, and EBA181) were associated with higher odds of cured infection. Using pooled sera, we analyzed their contribution to inhibition of merozoite invasion (Fig 1). Sera containing all six specific antibodies impaired *P*. *falciparum* invasion (*p*<0.05). Sera from both groups of patients containing five out of the six antigen-specific antibodies also impaired the parasite invasion (*p*<0.05). Although the invasion inhibition was statistically insignificant between five specific antibodies-containing sera from cured and recrudescent patients (*p* = 0.079), the invasion inhibition observed with cured sera was consistently higher than that observed with recrudescent sera.

## Discussion

Humoral immunity could predispose infected patients receiving drug therapy to a better disease resolution. Using sera from patients who (1) have subsequent recrudescent infection, or (2) were cured by Day 28 following treatment, we examined their naturally acquired immunity. In an earlier study using a slightly larger sera set [8], all cured patients developed antibodies against ring-infected erythrocyte surface antigen (RESA), compared to only 60% of recrudescent patients. Parasite growth was significantly lower in cultures with anti-RESA antibodies, suggesting that anti-malarial antibodies contribute to the therapeutic outcome following drug treatment. Similarly, other research groups have since showed the importance of host immunity in better treatment outcome [11, 12].

In this study, we further examined the antibody profile of the sera sets. Using our *P*. *falciparum* antigen library, we found that the antibody repertoire of both groups of sera was extremely broad and varied. In general, cured patients also had a greater (or similar) antibody response to the antigens in the library, compared to the recrudescent patients. With the exception of AMA-1, higher antibody response was not observed with recrudescent patients. Despite the higher antibody response to AMA-1, the patients had recrudescent infection, suggesting that the presence of AMA-1 antibody might be not sufficient to offer protection. This is in agreement with a recent study where AMA-1 vaccination has not demonstrated efficacy against blood stage model of human controlled malaria infection [13].

One of the main findings of this study is the differential antibody profile between the sera from cured and recrudescent patients. The proportion of cured patients with antibodies specific for EXP1, MSP3, GLURP, RAMA, SEA and EBA181 was higher than the proportion of recrudescent patients. The presence of these antibodies was associated with higher odds ratio of treatment cure. Although the proportion of cured patients with antibodies specific for SEA and EBA181 was not found to be statistically higher than the proportion of recrudescent patients after the *p* value was adjusted for multiple comparisons using Bonferroni correction, we have also included two antigens in our analysis. This is because (1) the analysis of sera containing specific antibodies against the four antigens (EXP1, MSP3, GLURP, RAMA) might be limited due to low serum sample size (*n* = 2), (3) both EBA181 and SEA could have a role in invasion and egress respectively [14, 15], and (3) Bonferroni correction is a highly stringent correction test. It is possible that, with larger serum sample size, the proportion of cured patients with antibodies specific for SEA and EBA181 could be statistically higher than the proportion of recrudescent patients. Antibody specific for RESA_aa501-1085_ was not found to be differentially induced in the two groups of patients, suggesting that this fragment of RESA does not contain the epitope for the antibodies that was identified in the earlier study [8] to contribute to the therapeutic response following drug treatment. One possible reason for this apparent disagreement is that perhaps the first half of the RESA protein (amino acid 1–500) might be needed as a scaffold to help form a conformational epitope consisting of the second half of the RESA protein (amino acid 501–1085).

The other main finding of the study is that pooled sera from cured patients significantly inhibited merozoite invasion at a greater extent as compared to pooled sera from recrudescent patients. Sera containing all six specific antibodies (EXP1, MSP3, GLURP, RAMA, SEA and EBA181) induced greater impairment of the reinvasion of clinical isolates of *P*. *falciparum*. While EXP1, a membrane glutathione S-transferase, has not been reported to be implicated in merozoite invasion, it is potently inhibited by artesunate [16]. The dual targeting of EXP1, by the immune system and by the antimalarial artesunate, is potentially an example where the host immunity and the drug therapy work synergistically to abrogate the function of the essential EXP1 and possibly eventually aid in disease resolution. MSP3, which is cleaved and shed at the tight junction between the invading merozoite and the RBC, has long been implicated in merozoite invasion [17, 18]. RAMA, a rhoptry-associated membrane antigen, binds specifically to RBCs and antibodies against RAMA inhibit merozoite invasion [19]. Anti-GLURP antibodies, from clinically immune Liberian adults, mediate a monocyte-dependent parasite growth inhibition [20]. SEA is involved in schizont egress and hence parasite multiplication [15]. Anti-EBA181 antibodies, from exposed Kenyan individuals, inhibits invasion [14]. These data hint at the possibility that merozoite reinvasion inhibition might be one of the mechanisms of action for the advantageous contributory role of the cured sera over the recrudescent sera in better disease resolution. We cannot conclusively deduce if the inhibition of invasion is due to a precedent inability to egress or an actual inability to invade the RBC. In addition, we do not exclude the possibility that there might be other yet to be identified *P*. *falciparum*-specific antibodies. Nevertheless, the lower rates of merozoite invasion observed with the sera from cured patients might help in containing the parasite levels and facilitate drug killing, suggesting a possible mechanism of action for their association with higher odds of treatment cure. This is particularly important in endemic areas where drug resistance with inefficient parasite clearance is evident.

The identification of a differential antibody profile that is associated with radical cure has implications in field settings in endemic areas. The presence of the panel of EXP1, MSP3, GLURP, RAMA, SEA and EBA181 antibodies could serve as an immune signature that could be relatively easy to detect upon first presentation in the field clinic. This provides critical information that would help in the design of the drug treatment regime better suited for the patients to achieve disease resolution. While there is an association between antibodies specific for EXP1, MSP3, GLURP, RAMA, SEA and EBA181and treatment cure, we caution against over interpretation as it does not necessarily indicate causation. Hence, it is of interest to further validate the association of the panel of EXP1, MSP3, GLURP, RAMA, SEA and EBA181 antibodies as an immune signature for predicting recrudescent infections in a larger cohort. In addition, due to the availability to small volume of the patients’ sera, more *in vitro* assays to purify single antibody against the six antigens for validating their activity in the merozoite invasion inhibition assay was not possible. Hence, it is also of interest to include this analysis using larger serum volumes and a larger cohort.

Our findings also have implications in the development for a malaria vaccine. A broad antibody repertoire may be essential for achieving optimal protection against malaria. This is consistent with our previous findings on human controlled malaria infection [9], where the antibody responses of the protected immunized individuals were broad and varied. Inhibition of merozoite invasion was more efficient in the presence of all six antibodies. The presence of all six antibodies was also associated with the lower odds of treatment failure. Hence, a multi-antigen vaccine might be more effective and could offer better protection against malaria.

Taken together, our findings suggest that antibodies specific against EXP1, MSP3, GLURP, RAMA, SEA and EBA181 in *P*. *falciparum* infections could assist anti-malarial drug treatment and contribute to the resolution of the malarial infection.

## Supporting Information

S1 Fig Transfection efficiency and antibody response. (A) and (B): Transfection efficiency was defined as Alexa Fluor 488-positive and PI-negative labelling, Q3. (A) Non-transfected cells, NT, where gates were applied to (B); (B) Pf antigen-transfected cells, in this case, Exp1, where the transfection efficiency was 86.5%. (C), (D) and (E): Antibody response, which was defined by Alexa Fluor 488-positive and PI-negative labelling (Q3), was gated on negative controls, (C) Pf antigen-transfected cells with serum from healthy volunteers and (D) non-transfected cells with patient sera. (E) Patient’s antibody response against Exp1 was 53.5%, as indicated in Q3. Exp1 reactivity for the patients was then normalised to the transfection efficiency, whereby (53.5/86.5)*100 gave an antigen reactivity of 61.8%.
(TIFF)

S1 Table Antibody repertoire of sera from cured and recrudescent patients. (DOCX)

S2 Table Specificity of antibodies in sera from cured and recrudescent patients. (DOCX)

S3 Table Differential antibody profile of sera obtained from cured and recrudescent patients with either severe or uncomplicated malaria. (DOCX)
