# Supplementary figures and images for: Neutralizing Antibodies against Plasmodium falciparum Associated with Successful Cure after Drug Therapy
Source: PLoS One. 2016 Jul 18;11(7):e0159347. doi: 10.1371/journal.pone.0159347 (PMC4948787; doi:10.1371/journal.pone.0159347)

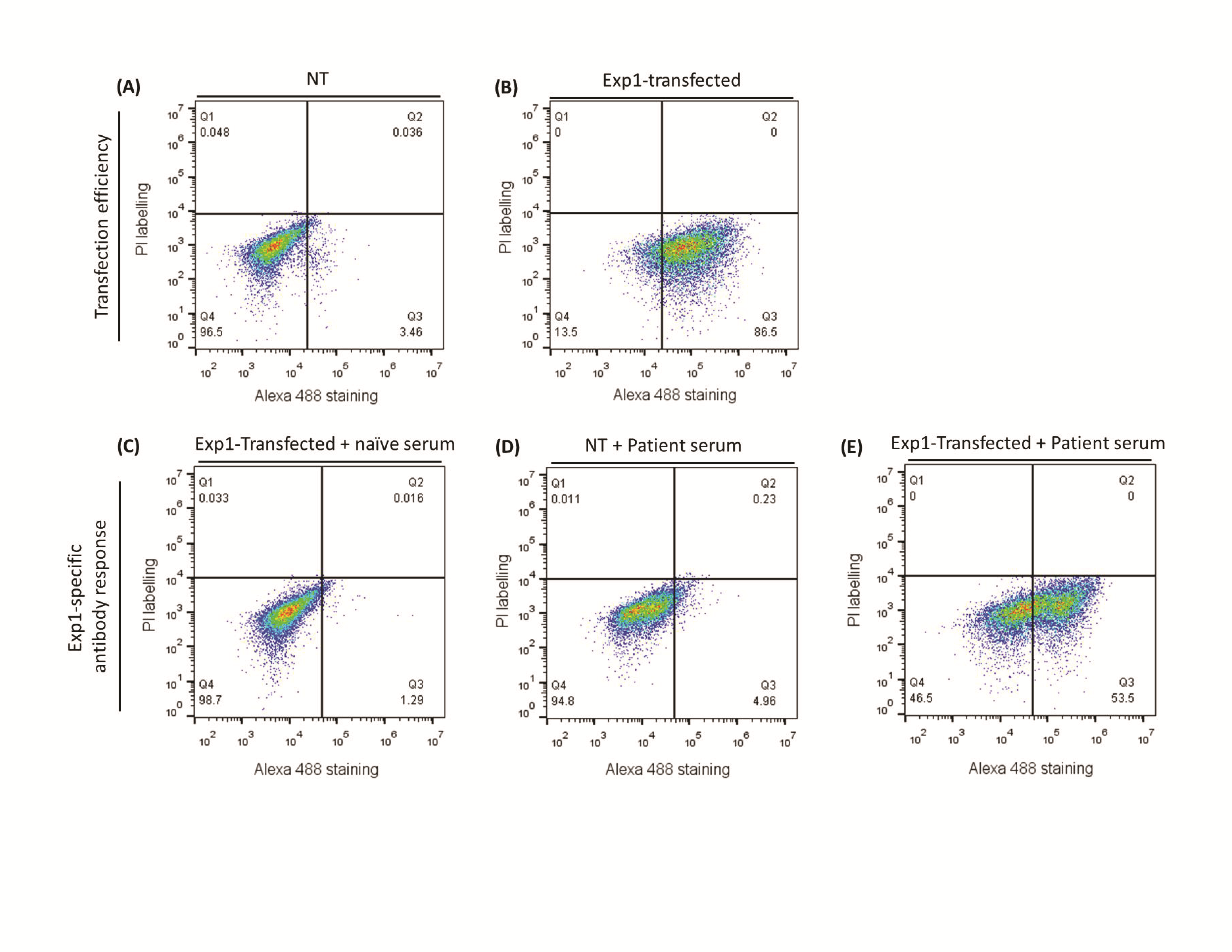

Supplement: S1 Fig — (A) and (B): Transfection efficiency was defined as Alexa Fluor 488-positive and PI-negative labelling, Q3. (A) Non-transfected cells, NT, where gates were applied to (B); (B) Pf antigen-transfected cells, in this case, Exp1, where the transfection efficiency was 86.5%. (C), (D) and (E): Antibody response, which was defined by Alexa Fluor 488-positive and PI-negative labelling (Q3), was gated on negative controls, (C) Pf antigen-transfected cells with serum from healthy volunteers and (D) non-transfected cells with patient sera. (E) Patient’s antibody response against Exp1 was 53.5%, as indicated in Q3. Exp1 reactivity for the patients was then normalised to the transfection efficiency, whereby (53.5/86.5)*100 gave an antigen reactivity of 61.8%. (TIFF) [file pone.0159347.s001.tiff]
