# Supplementary material for: Neutralizing Antibodies against Plasmodium falciparum Associated with Successful Cure after Drug Therapy
Source: PLoS One. 2016 Jul 18;11(7):e0159347. doi: 10.1371/journal.pone.0159347 (PMC4948787; doi:10.1371/journal.pone.0159347)
Supplement: S1 Table — (DOCX) [file pone.0159347.s002.docx]

S1 Table. Antibody repertoire of sera from pooled cured and recrudescent patients

| Gene name | plasmodb | Transfection  efficiency (%) | Cured patients | | Recrudescent patients | |
| --- | --- | --- | --- | --- | --- | --- |
|  |  |  | IgG | IgM | IgG | IgM |
| maebl | PF3D7_1147800 | 95 | 10.75 | 10.17 | 10.08 | < 10.00 |
| maebl | PF3D7_1147800 | 23 | 16.83 | < 10.00 | 11.17 | < 10.00 |
| EBL140 | PF3D7_1301600 | 55 | 23.83 | 39.00 | < 10.00 | < 10.00 |
| EXP1 | PF3D7_1121600 | 98 | 32.21 | < 10.00 | 16.34 | < 10.00 |
| EXP1 | PF3D7_1121600 | 87 | 53.21 | 47.49 | 20.89 | 32.51 |
| HPTM1 | PF3D7_0932800 | 96 | 0.09 | 10.81 | < 10.00 | < 10.00 |
| HPTM1 | PF3D7_0932800 | 93 | 12.73 | 10.07 | 10.83 | < 10.00 |
| LSA3 | PF3D7_0220000 | 78 | 95.75 | 58.71 | 96.21 | 43.43 |
| LSA3 | PF3D7_0220000 | 95 | 38.89 | 38.49 | 37.84 | < 10.00 |
| LSA3 | PF3D7_0220000 | 97 | 48.31 | 18.72 | 44.44 | < 10.00 |
| MSP3 | PF3D7_1035400 | 92 | 73.02 | 20.82 | 43.39 | 11.82 |
| MSP7 | PF3D7_1334800 | 89 | 36.67 | < 10.00 | 12.08 | < 10.00 |
| PF38 | PF3D7_0508000 | 61 | 35.17 | 13.00 | 15.00 | 12.00 |
| PF92 | PF3D7_1364100 | 38 | 49.57 | 24.14 | 50.00 | 15.87 |
| MSP2 | PF3D7_0206800 | 22 | 21.57 | 20.00 | 16.00 | < 10.00 |
| MSP2 | PF3D7_0206800 | 98 | 20.17 | < 10.00 | 14.05 | < 10.00 |
| PALPF3 | PF3D7_1340900 | 97 | < 10.00 | < 10.00 | < 10.00 | < 10.00 |
| PALPF3 | PF3D7_1340900 | 78 | < 10.00 | < 10.00 | < 10.00 | < 10.00 |
| MTRAP | PF3D7_1028700 | 95 | 12.20 | < 10.00 | 1.28 | 10.67 |
| MTRAP | PF3D7_1028700 | 98 | 15.72 | 14.51 | 14.43 | 11.49 |
| GLURP | PF3D7_1035300 | 91 | 76.79 | 66.26 | 43.83 | 35.87 |
| RAMA | PF3D7_1035300 | 78 | 42.53 | 26.89 | 15.21 | 13.55 |
| RHOPH1 | PF3D7_0220800 | 80 | 11.66 | < 10.00 | 12.51 | < 10.00 |
| RHOPH1 | PF3D7_0220800 | 78 | 10.57 | < 10.00 | 6.37 | < 10.00 |
| RHOPH3 | PF3D7_0905400 | 55 | < 10.00 | < 10.00 | < 10.00 | < 10.00 |
| PALPF4 | PF3D7_0505200 | 78 | < 10.00 | < 10.00 | < 10.00 | < 10.00 |
| PALPF2 | PF3D7_0418100 | 96 | < 10.00 | 10.28 | < 10.00 | < 10.00 |
| PALPF2 | PF3D7_0418100 | 89 | 15.73 | 30.09 | < 10.00 | < 10.00 |
| PALPF2 | PF3D7_0418100 | 40 | < 10.00 | < 10.00 | < 10.00 | < 10.00 |
| PfSEA | PF3D7_1021800 | 42 | 42.82 | 28.82 | 13.61 | 18.88 |
| TRAP | PF3D7_1335900 | 27 | 17.17 | 30.00 | 18.00 | 12.50 |
| TRAP | PF3D7_1335900 | 90 | 22.02 | 14.62 | 16.16 | 12.18 |
| EBL181 | PF3D7_0102500 | 55 | 23.13 | < 10.00 | 22.13 | < 10.00 |
| EBL181 | PF3D7_0102500 | 83 | 43.00 | 17.87 | 17.30 | 13.67 |
| PF92 | PF3D7_1364100 | 20 | < 10.00 | < 10.00 | < 10.00 | < 10.00 |
| Etramp14.2 | PF3D7_1476100 | 98 | < 10.00 | < 10.00 | < 10.00 | < 10.00 |
| HPTM3 | PF3D7_0627100 | 97 | 11.82 | 20.48 | 13.47 | 12.92 |
| HPTM3 | PF3D7_0627100 | 95 | < 10.00 | < 10.00 | < 10.00 | < 10.00 |
| RAMA | PF3D7_1035300 | 91 | 35.96 | 18.12 | 13.31 | 12.10 |
| RHOPH3 | PF3D7_0905400 | 71 | < 10.00 | < 10.00 | < 10.00 | < 10.00 |
| GLURP | PF3D7_1035300 | 93 | 50.63 | 30.53 | 25.63 | 24.94 |
| AMA-1 | PF3D7_1133400 | 70 | 43.27 | 24.08 | 70.83 | 16.83 |
| EBA175 | PF3D7_0731500 | 43 | 62.58 | 29.44 | 44.22 | 17.11 |
| EBA175 | PF3D7_0731500 | 79 | 45.25 | 17.67 | 30.44 | 14.92 |
| EBL140 | PF3D7_1301600 | 60 | 22.00 | 30.67 | < 10.00 | 15.83 |
| PF113 | PF3D7_1420700 | 58 | 14.33 | < 10.00 | < 10.00 | < 10.00 |
| RESA | PF3D7_0102200 | 47 | 26.67 | < 10.00 | 24.21 | < 10.00 |
| HPTM3 | PF3D7_0627100 | 38 | < 10.00 | < 10.00 | < 10.00 | < 10.00 |
| Hypothetical protein | PF3D7_1134300 | 92 | < 10.00 | < 10.00 | < 10.00 | < 10.00 |
| Rh5 | PF3D7_0424100 | 22 | 14.67 | 16.33 | 15.00 | 17.00 |
| RAP1 | PF3D7_1410400 | 21 | 18.33 | 13.54 | < 10.00 | 16.83 |
| RAP3 | PF3D7_0501500 | 20 | 32.5 | 23.50 | < 10.00 | 22.17 |
| PF12 | PF3D7_0612700 | 20 | < 10.00 | < 10.00 | < 10.00 | < 10.00 |
| PF41 | PF3D7_0404900 | 26 | < 10.00 | 26.67 | < 10.00 | 27.50 |
| HP4 | PF3D7_1404900 | 45 | < 10.00 | < 10.00 | < 10.00 | < 10.00 |
| PF45 | PF3D7_1346700 | 69 | 22.14 | 28.57 | < 10.00 | < 10.00 |
| MSPDBL1 | PF3D7_1035700 | 44 | 44.5 | 24.13 | 50 | 10.38 |
| EBA175III-V | PF3D7_0731500 | 91 | 19.98 | < 10.00 | 22.46 | < 10.00 |
| RON4 | PF3D7_1116000 | 83 | 28.6 | < 10.00 | 31.5 | < 10.00 |
| EBL140 | PF3D7_1301600 | 21 | < 10.00 | < 10.00 | < 10.00 | < 10.00 |
| PIESP1 | PF3D7_0310400 | 36 | < 10.00 | < 10.00 | < 10.00 | < 10.00 |
| GAMA | PF3D7_0828800 | 20 | < 10.00 | < 10.00 | < 10.00 | < 10.00 |
| Ripr | PF3D7_0323400 | 22 | < 10.00 | < 10.00 | < 10.00 | < 10.00 |
| Rh2a | PF3D7_1335400 | 30 | < 10.00 | < 10.00 | < 10.00 | < 10.00 |
| Rh2b | PF3D7_1335300 | 22 | < 10.00 | < 10.00 | < 10.00 | < 10.00 |
| Rh4 | PF3D7_0424200 | 21 | < 10.00 | < 10.00 | < 10.00 | < 10.00 |
| Rh5 | PF3D7_0424100 | 20 | < 10.00 | < 10.00 | < 10.00 | < 10.00 |
| RALP1 | PF3D7_0722200 | 30 | < 10.00 | < 10.00 | < 10.00 | < 10.00 |
| RON2 | PF3D7_1452000 | 34 | < 10.00 | < 10.00 | < 10.00 | < 10.00 |
| Pf52 | PF3D7_0404500 | 21 | 16.50 | 35.50 | < 10.00 | < 10.00 |
| Etramp4 | PF3D7_0423700 | 20 | < 10.00 | < 10.00 | < 10.00 | < 10.00 |
| HP3 | PF3D7_0911900 | 20 | 21.38 | 14.63 | 23.25 | < 10.00 |
| STARP | PF3D7_0702300 | 21 | < 10.00 | < 10.00 | < 10.00 | < 10.00 |
| PIESP1 | PF3D7_0310400 | 26 | < 10.00 | < 10.00 | < 10.00 | < 10.00 |
| MSP10 | PF3D7_0620400 | 20 | < 10.00 | < 10.00 | < 10.00 | < 10.00 |
| MSP7 | PF3D7_1335100 | 82 | 49.61 | < 10.00 | 52.61 | < 10.00 |
| MSRP1 | PF3D7_1335000 | 89 | < 10.00 | < 10.00 | < 10.00 | < 10.00 |
| AMA1 | PF3D7_1133400 | 40 | 27.17 | < 10.00 | 56.67 | < 10.00 |
| Hypothetical protein | PF3D7_0606800 | 36 | < 10.00 | < 10.00 | < 10.00 | < 10.00 |
| Pf113 | PF3D7_1420700 | 59 | 55.75 | < 10.00 | 59.5 | < 10.00 |
| Hypothetical protein | PF3D7_1136200 | 48 | < 10.00 | < 10.00 | < 10.00 | < 10.00 |
| Celtos | PF3D7_1216600 | 70 | < 10.00 | < 10.00 | < 10.00 | < 10.00 |
| ETRAMP10.3 | PF3D7_1016900 | 43 | < 10.00 | < 10.00 | < 10.00 | < 10.00 |
| MIF | PF3D7_1229400 | 88 | < 10.00 | < 10.00 | < 10.00 | < 10.00 |
