# Supplementary material for: Neutralizing Antibodies against Plasmodium falciparum Associated with Successful Cure after Drug Therapy
Source: PLoS One. 2016 Jul 18;11(7):e0159347. doi: 10.1371/journal.pone.0159347 (PMC4948787; doi:10.1371/journal.pone.0159347)
Supplement: S2 Table — (DOCX) [file pone.0159347.s003.docx]

S2 Table. Specificity of antibodies in sera from cured and recrudescent patients

| Gene name | plasmodb | Transfection  efficiency (%) | Cured patients with specific Ab (%) | | Recrudescent patients with specific Ab (%) | |
| --- | --- | --- | --- | --- | --- | --- |
|  |  |  | IgG | IgM | IgG | IgM |
| maebl | PF3D7_1147800 | 95 | 17.50 | 14.29 | 12.50 | 10.71 |
| maebl | PF3D7_1147800 | 23 | 12.50 | 10.71 | 7.50 | 3.57 |
| EBL140 | PF3D7_1301600 | 55 | 32.50 | 17.86 | 20.00 | 17.86 |
| EXP1 | PF3D7_1121600 | 98 | 40.00 | 32.14 | 10.00 | 0.00 |
| EXP1 | PF3D7_1121600 | 87 | 95.00 | 57.14 | 65.00 | 46.43 |
| HPTM1 | PF3D7_0932800 | 96 | 7.50 | 10.71 | 12.50 | 10.71 |
| HPTM1 | PF3D7_0932800 | 93 | 20.00 | 28.57 | 17.50 | 10.71 |
| LSA3 | PF3D7_0220000 | 78 | 92.50 | 82.14 | 52.50 | 32.14 |
| LSA3 | PF3D7_0220000 | 95 | 80.00 | 60.71 | 40.00 | 7.14 |
| LSA3 | PF3D7_0220000 | 97 | 70.00 | 53.57 | 25.00 | 7.14 |
| MSP3 | PF3D7_1035400 | 92 | 95.00 | 46.43 | 35.00 | 28.57 |
| MSP7 | PF3D7_1334800 | 89 | 55.00 | 42.86 | 10.00 | 7.14 |
| PF38 | PF3D7_0508000 | 61 | 62.50 | 50.00 | 47.50 | 10.71 |
| PF92 | PF3D7_1364100 | 38 | 57.50 | 39.29 | 30.00 | 35.71 |
| MSP2 | PF3D7_0206800 | 22 | 15.00 | 21.43 | 25.00 | 10.71 |
| MSP2 | PF3D7_0206800 | 98 | 32.50 | 39.29 | 5.00 | 7.14 |
| PALPF3 | PF3D7_1340900 | 97 | 10.00 | 7.14 | 10.00 | 14.29 |
| PALPF3 | PF3D7_1340900 | 78 | 0.00 | 0.00 | 10.00 | 14.29 |
| MTRAP | PF3D7_1028700 | 95 | 27.50 | 14.29 | 22.50 | 35.71 |
| MTRAP | PF3D7_1028700 | 98 | 37.50 | 39.29 | 22.50 | 28.57 |
| GLURP | PF3D7_1035300 | 91 | 97.50 | 82.14 | 77.50 | 35.71 |
| RAMA | PF3D7_1035300 | 78 | 85.00 | 32.14 | 22.50 | 25.00 |
| RHOPH1 | PF3D7_0220800 | 80 | 20.00 | 32.14 | 12.50 | 17.86 |
| RHOPH1 | PF3D7_0220800 | 78 | 22.50 | 28.57 | 5.00 | 14.29 |
| RHOPH3 | PF3D7_0905400 | 55 | 12.50 | 25.00 | 5.00 | 3.57 |
| PALPF4 | PF3D7_0505200 | 78 | 12.50 | 25.00 | 2.50 | 3.57 |
| PALPF2 | PF3D7_0418100 | 96 | 10.00 | 17.86 | 27.50 | 25.00 |
| PALPF2 | PF3D7_0418100 | 89 | 12.50 | 21.43 | 22.50 | 21.43 |
| PALPF2 | PF3D7_0418100 | 40 | 0.00 | 7.14 | 5.00 | 3.57 |
| PfSEA | PF3D7_1021800 | 42 | 85.00 | 57.14 | 25.00 | 25.00 |
| TRAP | PF3D7_1335900 | 27 | 35.00 | 32.14 | 35.00 | 39.29 |
| TRAP | PF3D7_1335900 | 90 | 12.50 | 42.86 | 20.00 | 32.14 |
| EBL181 | PF3D7_0102500 | 55 | 47.50 | 46.43 | 20.00 | 28.57 |
| EBL181 | PF3D7_0102500 | 83 | 55.00 | 28.57 | 22.50 | 28.57 |
| PF92 | PF3D7_1364100 | 20 | 10.00 | 7.14 | 0.00 | 21.43 |
| Etramp14.2 | PF3D7_1476100 | 98 | 15.00 | 7.14 | 7.50 | 7.14 |
| HPTM3 | PF3D7_0627100 | 97 | 57.50 | 60.71 | 40.00 | 53.57 |
| HPTM3 | PF3D7_0627100 | 95 | 2.50 | 0.00 | 0.00 | 0.00 |
| RAMA | PF3D7_1035300 | 91 | 45.00 | 46.43 | 35.00 | 28.57 |
| RHOPH3 | PF3D7_0905400 | 71 | 7.50 | 10.71 | 2.50 | 0.00 |
| GLURP | PF3D7_1035300 | 93 | 92.50 | 82.14 | 85.00 | 75.00 |
| AMA-1 | PF3D7_1133400 | 70 | 25.00 | 39.29 | 10.00 | 17.86 |
| EBA175 | PF3D7_0731500 | 43 | 35.00 | 67.86 | 60.00 | 57.14 |
| EBA175 | PF3D7_0731500 | 79 | 22.50 | 46.43 | 15.00 | 28.57 |
| EBL140 | PF3D7_1301600 | 60 | 15.00 | 35.71 | 15.00 | 28.57 |
| PF113 | PF3D7_1420700 | 58 | 5.00 | 25.00 | 15.00 | 0.00 |
| RESA | PF3D7_0102200 | 47 | 17.50 | 32.14 | 10.00 | 3.57 |
| HPTM3 | PF3D7_0627100 | 38 | 2.50 | 3.57 | 0.00 | 0.00 |
| Hypothetical protein | PF3D7_1134300 | 92 | 0.00 | 0.00 | 0.00 | 0.00 |
| Rh5 | PF3D7_0424100 | 22 | 25.00 | 25.00 | 32.50 | 35.71 |
| RAP1 | PF3D7_1410400 | 21 | 10.00 | 17.86 | 12.50 | 17.86 |
| RAP3 | PF3D7_0501500 | 20 | 42.50 | 21.43 | 30.00 | 25.00 |
| PF12 | PF3D7_0612700 | 20 | 0.00 | 0.00 | 0.00 | 0.00 |
| PF41 | PF3D7_0404900 | 26 | 15.00 | 17.86 | 12.50 | 25.00 |
| HP4 | PF3D7_1404900 | 45 | 10.00 | 3.57 | 2.50 | 3.57 |
| PF45 | PF3D7_1346700 | 69 | 30.00 | 21.43 | 10.00 | 7.14 |
| MSPDBL1 | PF3D7_1035700 | 44 | 30.00 | 32.14 | 15.00 | 28.57 |
| EBA175III-V | PF3D7_0731500 | 91 | 30.00 | 17.86 | 5.00 | 10.71 |
| RON4 | PF3D7_1116000 | 83 | 25.00 | 21.43 | 5.00 | 10.71 |
| EBL140 | PF3D7_1301600 | 21 | 10.00 | 3.57 | 12.50 | 7.14 |
| PIESP1 | PF3D7_0310400 | 36 | 2.50 | 3.57 | 5.00 | 0.00 |
| GAMA | PF3D7_0828800 | 20 | 2.50 | 0.00 | 10.00 | 0.00 |
| Ripr | PF3D7_0323400 | 22 | 2.50 | 0.00 | 0.00 | 0.00 |
| Rh2a | PF3D7_1335400 | 30 | 0.00 | 10.71 | 5.00 | 0.00 |
| Rh2b | PF3D7_1335300 | 22 | 0.00 | 0.00 | 0.00 | 0.00 |
| Rh4 | PF3D7_0424200 | 21 | 2.50 | 0.00 | 5.00 | 0.00 |
| Rh5 | PF3D7_0424100 | 20 | 0.00 | 0.00 | 7.50 | 0.00 |
| RALP1 | PF3D7_0722200 | 30 | 0.00 | 0.00 | 0.00 | 0.00 |
| RON2 | PF3D7_1452000 | 34 | 0.00 | 0.00 | 0.00 | 0.00 |
| Pf52 | PF3D7_0404500 | 21 | 7.50 | 10.71 | 15.00 | 7.14 |
| Etramp4 | PF3D7_0423700 | 20 | 5.00 | 10.71 | 2.50 | 0.00 |
| HP3 | PF3D7_0911900 | 20 | 50.00 | 42.86 | 10.00 | 7.14 |
| STARP | PF3D7_0702300 | 21 | 12.50 | 17.86 | 5.00 | 0.00 |
| PIESP1 | PF3D7_0310400 | 26 | 10.00 | 14.29 | 2.50 | 0.00 |
| MSP10 | PF3D7_0620400 | 20 | 2.50 | 0.00 | 2.50 | 0.00 |
| MSP7 | PF3D7_1335100 | 82 | 42.50 | 28.57 | 10.00 | 0.00 |
| MSRP1 | PF3D7_1335000 | 89 | 0.00 | 0.00 | 0.00 | 0.00 |
| AMA1 | PF3D7_1133400 | 40 | 7.50 | 14.29 | 2.50 | 0.00 |
| Hypothetical protein | PF3D7_0606800 | 36 | 5.00 | 0.00 | 0.00 | 0.00 |
| Pf113 | PF3D7_1420700 | 59 | 70.00 | 64.29 | 17.50 | 17.86 |
| Hypothetical protein | PF3D7_1136200 | 48 | 2.50 | 0.00 | 2.50 | 0.00 |
| Celtos | PF3D7_1216600 | 70 | 0.00 | 0.00 | 20.00 | 14.29 |
| ETRAMP10.3 | PF3D7_1016900 | 43 | 0.00 | 0.00 | 2.50 | 0.00 |
| MIF | PF3D7_1229400 | 88 | 0.00 | 0.00 | 0.00 | 0.00 |
