# Supplementary material for: Neutralizing Antibodies against Plasmodium falciparum Associated with Successful Cure after Drug Therapy
Source: PLoS One. 2016 Jul 18;11(7):e0159347. doi: 10.1371/journal.pone.0159347 (PMC4948787; doi:10.1371/journal.pone.0159347)
Supplement: S3 Table — (DOCX) [file pone.0159347.s004.docx]

S3 Table. Differential antibody profile of sera obtained from cured and recrudescent patients with either severe or uncomplicated malaria.

|  |  |  | Severe malaria | | | | Uncomplicated malaria | | | |
| --- | --- | --- | --- | --- | --- | --- | --- | --- | --- | --- |
|  |  |  | Cured patients with specific Ab (%) | | Recrudescent patients with specific Ab (%) | | Cured patients with specific Ab (%) | | Recrudescent patients with specific Ab (%) | |
| Gene name | plasmodb | Transfection efficiency (%) | IgG | IgM | IgG | IgM | IgG | IgM | IgG | IgM |
| EXP1 | PF3D7_1121600 | 87 | 100 | 69.2 | 69.2 | 69.2 | 92.5 | 62.9 | 46.7 | 26.7 |
| MSP3 | PF3D7_1035400 | 92 | 100 | 30.8 | 30.8 | 30.8 | 92.5 | 33.3 | 60 | 26.7 |
| GLURP | PF3D7_1035300 | 91 | 100 | 69.2 | 76.9 | 7.7 | 96.3 | 81.5 | 86.7 | 46.7 |
| RAMA | PF3D7_1035300 | 78 | 92.3 | 7.7 | 38.5 | 7.7 | 81.5 | 29.6 | 26.7 | 40 |
| PfSEA | PF3D7_1021800 | 42 | 69.2 | 7.7 | 53.8 | 7.7 | 92.6 | 33.3 | 60 | 40 |
| EBA181 | PF3D7_0102500 | 83 | 76.9 | 23.1 | 30.8 | 38.5 | 40.7 | 22.2 | 26.7 | 20 |
